# Sitagliptin-induced Pancreatitis: Chronic Use Would Not Spare You the Complication

**DOI:** 10.7759/cureus.7389

**Published:** 2020-03-24

**Authors:** Talal Alkayali, Juan Ricardo, Kafayat Busari, Ibrahim Saad

**Affiliations:** 1 Internal Medicine, Florida State University College of Medicine Internal Medicine Residency Program at Sarasota Memorial Hospital, Sarasota, USA

**Keywords:** acute pancreatitis, sitagliptin, dipeptidyl peptidase-4 inhibitor, medication-induced pancreatitis

## Abstract

Medication-induced pancreatitis is an overlooked cause of acute pancreatitis. We present an 81-year-old male patient with acute sharp epigastric pain radiating to his back, who was found to have lipase of more than 30,000 U/L. The patient denied current alcohol use. Abdominal ultrasound and abdominal computed tomography scan revealed no gallstones or biliary duct abnormalities. The patient had been taking sitagliptin for eight years. Supportive treatment with intravenous fluids, pain medications, and early feeding adequately treated his disease. With our case, we aim to increase awareness of sitagliptin-induced pancreatitis, regardless of the duration of use.

## Introduction

Acute pancreatitis is the most common gastrointestinal pathology requiring hospital admission [1,2]. From all the studied causes of acute pancreatitis, drug-induced acute pancreatitis is an uncommon etiology when compared to obstructing gallstones or alcohol use, accounting for 0.1% to 2% of identified causes [2,3]. Sitagliptin is an oral dipeptidyl peptidase-4 (DPP-4) inhibitor used to treat diabetes mellitus. This medication inhibits DPP-4, an enzyme that inactivates glucagon-like peptide-1 (GLP-1), leading to prolongation of the half-life of GLP-1 in the body. GLP-1 stimulates glucose-dependent insulin release from the pancreatic islets leading to decreased blood glucose levels, slowing gastric emptying, and inhibiting inappropriate post-meal glucagon release [4].

## Case presentation

An 81-year-old male patient with a history of diabetes mellitus type 2 presented to the emergency department with severe sharp epigastric pain for a few hours after eating. The pain started suddenly, radiated to his back, worsened with movements, was without alleviating factors, and was associated with nausea. He denied any vomiting, diarrhea, fevers, melena, or chest pain. The patient denied alcohol use, changes in prescriptions, and use of herbal or over-the-counter medications. On review of his medications, it was noted he had been taking sitagliptin 100 mg daily for many years. He was also on aspirin, atorvastatin, tamsulosin, pioglitazone, donepezil/memantine, metoprolol, and insulin degludec.

Examination revealed a hypertensive elderly male patient in distress. The abdomen was soft with epigastric tenderness noted on palpation. No jaundice, rebound tenderness, rigidity, or ascites noted. Murphy’s sign was negative, and the remaining physical exam was otherwise unremarkable.

**Figure 1:**
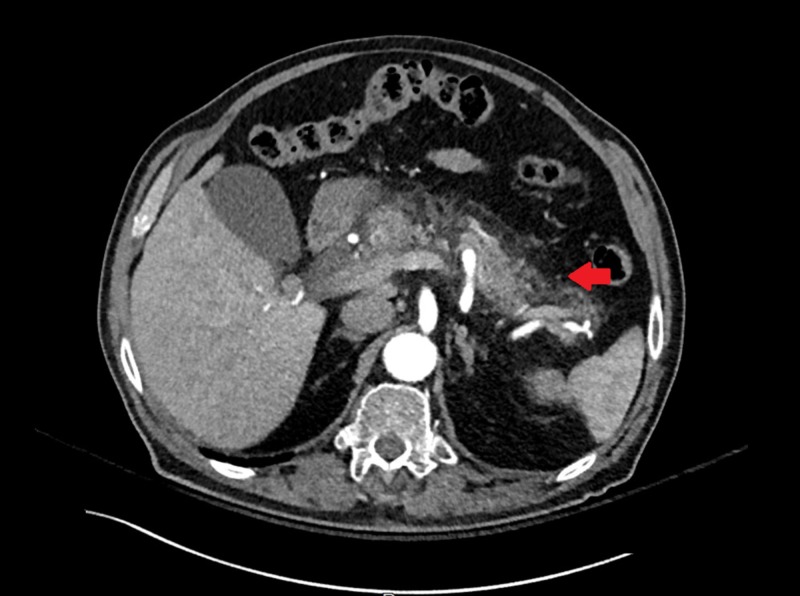
Enlarged pancreas with diffuse edema and peripancreatic inflammation

Blood workup was pertinent for an elevated lipase of more than 30,000 U/L and a slight elevation in aspartate aminotransferase (56 U/L). Alanine aminotransferase, alkaline phosphatase, bilirubin, hemogram, creatinine, blood urea nitrogen, calcium, and triglycerides were normal. Liver ultrasound showed no liver or biliary duct abnormalities, no signs of gallstones, sludge, or wall thickening. An abdominal computerized tomography scan showed an enlarged pancreas with diffuse edema and peripancreatic inflammation consistent with acute pancreatitis, but no biliary or pancreatic duct dilatation or filling defects (Figure 1).

The patient was started on intravenous fluids, nothing per mouth, and hydromorphone. His home medications were continued, except for sitagliptin, which was held on admission. He showed improvement in the following 24 hours and was able to tolerate oral intake. His lipase trended down, and serum IgG4 was normal. After two days, he was noted to have a sudden increase in his liver function tests (LFTs; Table 1).

**Table 1:** Laboratory values WBC, white blood count; HG, hemoglobin; ALP, alkaline phosphatase; AST, aspartate transaminase; ALT, alanine transaminase; T. bilirubin, total bilirubin; D. bilirubin, direct bilirubin; Cr, creatinine; BUN, blood urea nitrogen; TG, triglyceride.

	Day 1	Day 2	Day 3	Day 4	Day 5	Day 6	Day 25
WBC (4.5-11 10^3^/µL)	6.7	7.3	-	-	9.9	8.8	5.0
HG (11.6-16.3 g/dL)	12.0	12.0	-	-	10.0	9.7	12.4
Platelet (150-400 10^3^/µL)	160	154	-	-	137	150	288
Lipase (73-393 U/L)	>30,000	14,396	6,695	1,027	429	-	310
ALP (26-162 U/L)	95	131	149	147	133	126	108
AST (15-37 U/L)	56	88	180	97	63	52	32
ALT (16-61 U/L)	52	100	153	119	85	74	43
T. bilirubin (0.2-1.3 mg/dL)	0.5	0.5	2.6	1.2	1.2	0.8	0.5
D. bilirubin (0-0.3 mg/dL)	0.3	-	1.6	0.6	-	-	-
Cr (0.70-1.30 mg/dL)	0.97	0.77	0.70	0.70	0.64	0.74	0.87
BUN (8-23 mg/dL)	10	10	8	8	9	9	21
Calcium (8.3-9.9 mg/dL)	8.4	-	-	-	-	-	-
TG (40-199 mg/dL)	76	-	-	-	-	-	-

Magnetic resonance cholangiopancreatography did not show any liver or biliary duct abnormalities. There was an incidental finding of iron deposition in the liver and spleen, prompting questionable hemochromatosis; however, iron studies and HFE gene were normal. The patient continued to improve, and lipase and LFTs normalized with supportive treatment. Prior to discharge, he was instructed to avoid the use of sitagliptin indefinitely.

## Discussion

Acute pancreatitis is a sudden inflammation of the pancreas. The course of the disease can range from a mild presentation of abdominal pain with nausea and vomiting to local pancreatic complications like the formation of peripancreatic fluid collections, pseudocysts, necrosis, and even systemic multi-organ failure [3]. Mortality for mild acute pancreatitis is estimated to be less than 1%, but if multiorgan failure develops, mortality can increase to 30% [3,5]. Gallstones and alcohol are the two most commonly identified causes. Other less common etiologies include medications, hypertriglyceridemia, hypercalcemia, idiopathic, trauma, endoscopic retrograde cholangiopancreatography induced, scorpion venom, and cystic fibrosis [1-3].

Since sitagliptin became available in the market, multiple studies and clinical trials were conducted to investigate its relationship to acute pancreatitis, and results have been conflicting; however, recently, sitagliptin has been identified as a possible agent to cause pancreatitis [3,4,6-11].

Preclinical data on sitagliptin effects on the pancreas histology in animals showed an association with acute pancreatitis. It is suggested that increased exposure to GLP-1 leads to increased pancreatic ductal turnover, ductal metaplasia, and inflammation and may accelerate the development of dysplastic lesions when already present [12,13]. However, other studies on animals with and without diabetes did not show any associations with pancreatic diseases in up to two years of follow-up [14].

Williams-Herman et al. evaluated a 12-pooled analysis on the safety of sitagliptin after two years of treatment, reporting no association between sitagliptin and acute pancreatitis [15]. Nonetheless, recent meta-analysis and case reports have supported the relationship between sitagliptin and pancreatic disorders [3,4,6-11]. In the Trial Evaluating Cardiovascular Outcomes with Sitagliptin Study, the association between sitagliptin and pancreatic disease suggested a small absolute increased risk for pancreatitis [7]. Multiple case reports also reported similar findings with even longer exposure to this medication in up to three years [9].

No time frame was proposed to indicate a clear-cut relationship. The patient had been taking sitagliptin for more than eight years, and yet he still developed this adverse effect. Other reports have shown patients presenting with mild cases of pancreatitis to severe cases of necrotizing pancreatitis, which has a higher mortality risk [4,10,11]. Further studies are required to shed more light on the association of DPP-4 inhibitors and pancreatic disorders.

## Conclusions

Medication-induced pancreatitis is an important etiology to be recognized early on to reduce unnecessary testing, imaging, and recurrence rates by limiting exposure. Providers should consider DPP-4 inhibitors as a cause of acute pancreatitis, even when patients have been on these medications for a prolonged time.
